# Genome-wide identification and expression of GRAS gene family members in cassava

**DOI:** 10.1186/s12870-020-2242-8

**Published:** 2020-01-29

**Authors:** Zhongying Shan, Xinglu Luo, Meiyan Wu, Limei Wei, Zhupeng Fan, Yanmei Zhu

**Affiliations:** 1 Agricultural College, Guangxi University, Nanning, 530005 China; 2 State Key Laboratory for Conservation and Utilization of Subtropical Agro-Bioresources, Nanning, 530004 China; 3 College of Ecology and Garden Architecture, Dezhou University, Dezhou, 253023 China

**Keywords:** Cassava, *GRAS* genes, Gene expression, Abiotic stress

## Abstract

**Background:**

Cassava is highly tolerant to stressful conditions, especially drought stress conditions; however, the mechanisms underlying this tolerance are poorly understood. The GRAS gene family is a large family of transcription factors that are involved in regulating the growth, development, and stress responses of plants. Currently, GRAS transcription factors have not been systematically studied in cassava, which is the sixth most important crop in the world.

**Results:**

Seventy-seven *MeGRAS* genes were identified from the cassava genome database. Phylogenetic analysis revealed that the MeGRAS proteins could be divided into 14 subfamilies. The gene structure and motif compositions of the proteins were considerably conserved within the same subfamily. Duplication events, particularly segmental duplication, were identified as the main driving force for *GRAS* gene expansion in cassava. Global expression analysis revealed that *MeGRAS* genes exhibited similar or distinct expression profiles within different tissues among different varieties. Moreover, qRT-PCR analysis revealed the expression patterns of *MeGRAS* genes in response to abiotic stress (drought, salt, cold, and H_2_O_2_), and the results suggest that these genes may have multiple functions.

**Conclusion:**

This study is the first to provide comprehensive information on GRAS gene family members in cassava. The data will increase our understanding of both the molecular basis and the effects of *GRAS* genes. In addition, the results will contribute further to identifying the responses to various environmental conditions and provide insights into the potential functions of *GRAS* genes.

## Background

Cassava (*Manihot esculenta* Crantz) is the sixth most important cash and food crop in Africa, Asia, Latin America, and the Caribbean. Cassava is cultivated for its starchy roots, which are used for food and many products. Owing to its inherent tolerance to stressful environments and the minimal care required, cassava is often considered a food security source against famine where other food crop species would fail. Under optimal environmental conditions, the energy production of cassava is greater than that of most other major staple food crop species [1].

With the advent of next-generation sequencing technology, a large number of genomic, transcriptomic, proteomic, and metabolomic data have been generated, providing a great opportunity for the development of metabolic engineering [2–10]. High-quality genome sequences of cassava have recently become available, which has greatly increased the understanding of the biological processes and molecular/cellular mechanisms in cassava [2, 3].

Transcription factors play regulatory roles in multiple physiological processes in higher plants. Transcription factors act as a switches of gene regulation; promote or inhibit the functional expression of specific genes; and are involved in maintaining the growth, development, and stress responses of plants [11, 12]. The term “GRAS” is derived from the first three transcription factors identified in this family: gibberellic acid insensitive (GAI), repressor of GAI (RGA), and scarecrow (SCR) [13]. Typically, GRAS proteins exhibit some C-terminal homology but diversification in sequence and length at their N-termini [13]. The leucine heptad repeat (LHR) I, VHIID, LHR II, PFYRE and SAW motifs are conserved, which is conducive to the function of proteins within the C-terminal region [13, 14]. The structure of VHIID as well as its two flanking regions (LHRs and LER II) is important for protein-protein interactions. Mutations in the PFYRE and SAW motifs result in distinct phenotypic abnormalities in *Arabidopsis* [15–17]. The N-termini of the GRAS proteins are diverse; however, the DELLA and TVHYNP motifs are conserved within the DELLA subfamily in the N-terminal region.

*GRAS* genes have recently been studied in various plant species, i.e., *Arabidopsis* [18], rice [18], Chinese cabbage [19], *Populus* [20], *Prunus mume* [21], tobacco [22], castor bean [23], grapevine [24], *Medicago truncatula* [25, 26], maize [27], *Malus domestica* [28], pepper [29], and tea plant [30]. According to previous studies in *Arabidopsis* and rice [18], the GRAS family members can be classified into eight subfamilies: the DELLA, HAM, LAS, PAT1, SCR, SHR, SCL3, and LISCL subfamilies. However, the number of subfamilies ranges from 8 to 16 in other plant species. GRAS proteins function in various physiological processes during plant growth and development. Considering the highly different amino acid (aa) sequences between each subfamily, each subfamily may have distinct functions. For example, DELLA members mainly function as repressors of gibberellin (GA) signalling [15, 16, 31–33]. The *HAIRY MERISTEM* gene from the HAM subfamily controls shoot meristem maintenance by mediating signals from differentiating cells [34]. Three genes (*MOC1*, *LS*, and *LAS*) from the LAS subfamily play important roles in axillary meristem initiation [35–37]. Three *Arabidopsis* genes (*PAT1*, *SCL5*, and *SCL21*) from the PAT1 subfamily are positive regulators of phytochrome-A signal transduction [38, 39], whereas *SCL13* from the same subfamily is involved mainly in phytochrome-B signal transduction [40]. SCR and SHR form an SCR/SHR complex, which plays an essential role in root and shoot radial organization [41–43]. SCL3 acts downstream of the GA/DELLA and SCR/SHR pathways and controls GA homeostasis during root development [44]. Moreover, the *LISCL* gene from the LISCL subfamily is involved in the microsporogenesis of anthers [45].

Until recently, the GRAS gene family in cassava has not been characterized. Thus, in the present study, members of the GRAS gene family were identified from a previous cassava genomic database. A phylogenetic tree was constructed, conversed motif and gene exon/intron structural analyses were performed, *MeGRAS* genes were mapped onto the cassava chromosomes, and *cis*-elements of *MeGRAS* genes and interaction network of MeGRAS proteins were analyzed. In addition, the expression patterns of the *MeGRAS* genes among different cassava varieties and different tissues were surveyed via available transcriptome data, and the expression patterns in response to various abiotic stresses were investigated via qRT-PCR in cassava. This research is the first to provide evidence concerning the cassava *GRAS* gene family, which may help elucidate the molecular mechanisms underlying stress responses in cassava.

## Results

### Identification of the *GRAS* gene family members in cassava

A total of 77 non-redundant *MeGRAS* genes were confirmed and used for subsequent analyses (Additional file 1: Table S1). With the exception of MeGRAS4, whose GRAS domain was divided into two parts by a sequence of 15 aas, all of the MeGRAS proteins contained a complete GRAS domain (PF03514). Three of the MeGRAS members (MeGRAS12, 72, and 73) had a DELLA domain (PF12041) and were thus considered DELLA proteins (Additional file 1: Table S2). The protein properties and subcellular localization were analysed, and the results are summarized in Additional file 1: Table S3. The length and molecular mass of the MeGRAS proteins varied greatly, with lengths ranging from 230 to 829 aas and molecular weights (MWs) ranging from 26.08 to 89.79 kDa. The average theoretical isoelectric point (pI) was 5.7, suggesting that most MeGRAS proteins were weakly acidic. With the exception of eight MeGRAS proteins (MeGRAS11, 19, 31, 34, 55, 63, 71, and 74), which were stable, all the MeGRAS proteins were considered unstable. The majority of the MeGRAS proteins contained a high percentage of aliphatic aas, with a predicted aliphatic index ranging from 68.16 to 99.77. Owing to their relatively low average hydropathy (GRAVY) value (< 0), all MeGRASs were predicted to be hydrophilic. It was predicted that 67.53% of the MeGRAS proteins localize to the nucleus. There were no transmembrane helices within the MeGRAS proteins except within GRAS21, which contained one helix that is targeted to the inside to the outside of cell membranes. The secondary structure prediction indicated that alpha helices and random coils were dominant in all of the MeGRAS aa sequences, followed by extended strands and beta turns, with average incidences of 44.90, 40.73, 10.11, and 4.26%, respectively (Additional file 1: Table S4).

### Phylogenetic analysis of the MeGRAS family

**Fig. 1:**
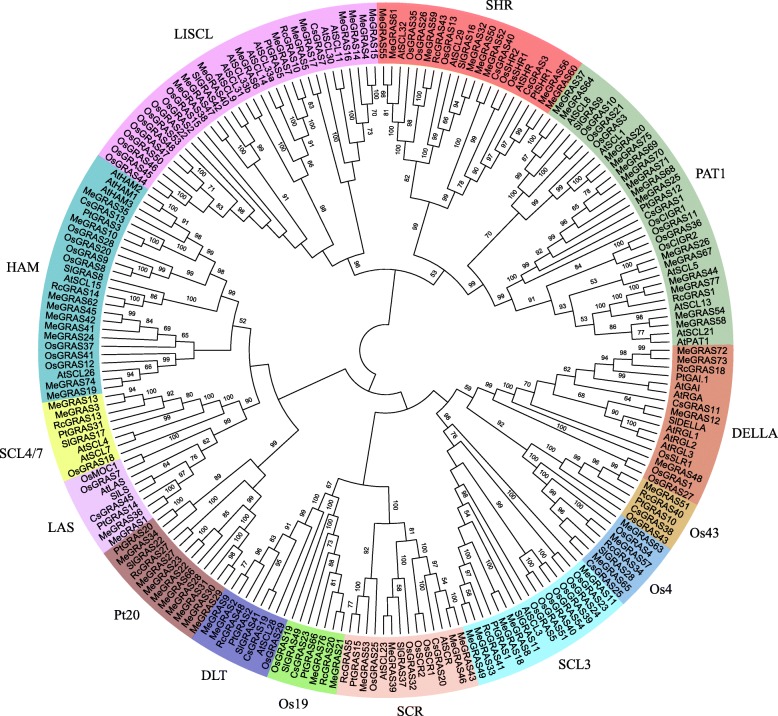
Phylogenetic analysis of GRAS proteins. The phylogenetic tree was constructed via the maximum likelihood method by MEGA-X. Subfamilies are indicated by different colours

**Fig. 2:**
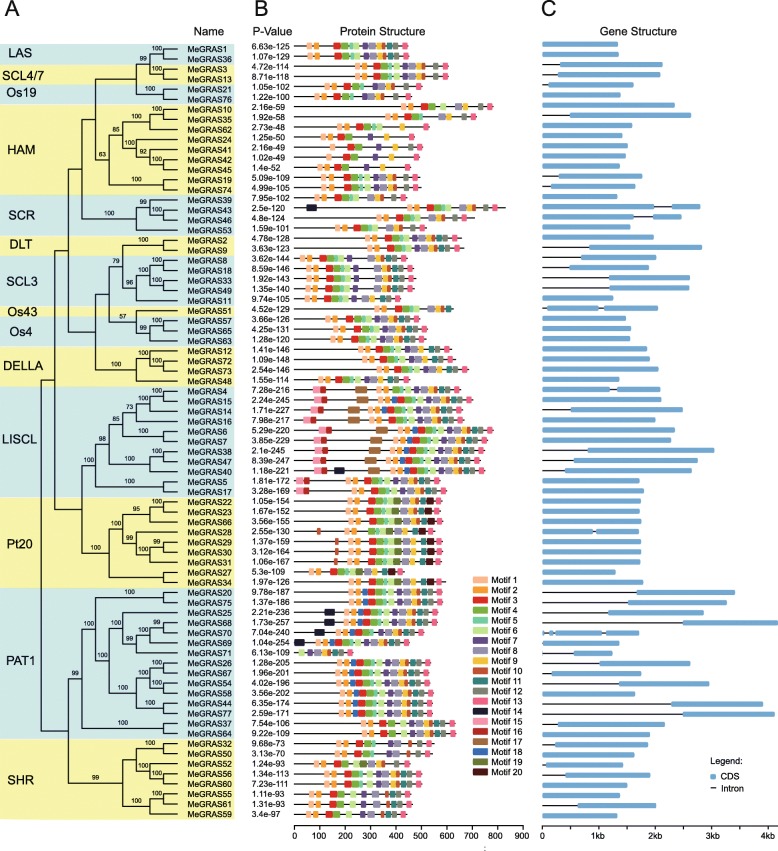
Distribution of conserved motifs within GRAS proteins in cassava. (**a**) The phylogenetic tree was constructed by MEGA-X with the neighbor-joining method. The same subfamilies are marked in yellow or light blue. (**b**) Motif distribution of GRAS proteins. The different motifs are indicated by different colours for motifs 1–20, and the combined *P*-values are shown on the left side of the figure. The same colour within different proteins refers to the same motif. The structural features of the 20 motifs are listed in Additional file 1: Table S7. (**c**) Structures of the 77 putative cassava *GRAS* genes. The exons and introns are represented by blue boxes and black lines, respectively

To uncover the evolutionary relationships of the GRAS gene family in cassava and to help in their classification, a total of 211 GRAS proteins, comprising 77 from cassava, 33 from *Arabidopsis*, 50 from rice, 13 from castor bean, 13 from *Populus*, 13 from tomato, and 12 from tea plant (Additional file 1: Table S5) [18, 20, 23, 29, 48], were performed to construct an unrooted phylogenetic tree using maximum likelihood (ML) method by MEGA-X (Fig. 1). Fourteen subfamilies were identified, including 15, 11, 9, 9, 8, 4, 4, 4, 3, 3, 2, 2, 2, and 1 MeGRAS members in the PAT1, LISCL, Pt20, HAM, SHR, SCL3, DELLA, SCR, DTL, Os4, LAS, SCL4/7, Os19, and Os43 subfamilies, respectively (Fig. 1, Fig. 2a).

### Motif composition and gene structure of MeGRAS family members

To investigate the structural features of cassava GRASs further, the protein conserved motifs and gene intron/exon distributions were analysed. A total of 20 conserved motifs (referred to as motifs 1–20) were identified by MEME (http://meme-suite.org/tools/meme), with more motifs located within the C-terminal region than within the N-terminal region (Fig. 2b); the features of these protein motifs are listed in Additional file 1: Table S6. The motifs from the same subfamily display nearly similar patterns. For example, members of SCL3 had the same motifs. This discovery provides additional evidence to support the close evolutionary relationship of MeGRAS members in the same subfamily. The motifs were matched with the corresponding GRAS domain. Motifs 1 and 2 were in the LHRI domain within the N-terminal region, followed by motifs 3, 4, and 5 in the VHIID domain; motifs 6 and 7 in the LHRII domain; motifs 8, 9, and 10 in the PFYRE domain; and motifs 12 and 13 in the SAW domain within the C-terminal region (Fig. 2b). Gene structural diversity is an important part of gene family evolution and further supports phylogenetic groupings [46]. In the present study, the number of introns varied from one to three (Fig. 2c). Among the 77 *MeGRAS* genes, 36 had introns, 34 had only one intron, and 41 had no introns. Generally, *MeGRAS* genes within the same subfamily in the phylogenetic tree had similar exon-intron structures. The LAS, DELLA, and Os4 subfamilies had no introns, and the SCL4/7, Os43, and PAT1 subfamilies had 1, 2, and 0–3 introns, respectively. The other subfamilies had 0–1 introns.

### Chromosomal localization and gene duplication analysis of *MeGRAS* genes

**Fig. 3:**
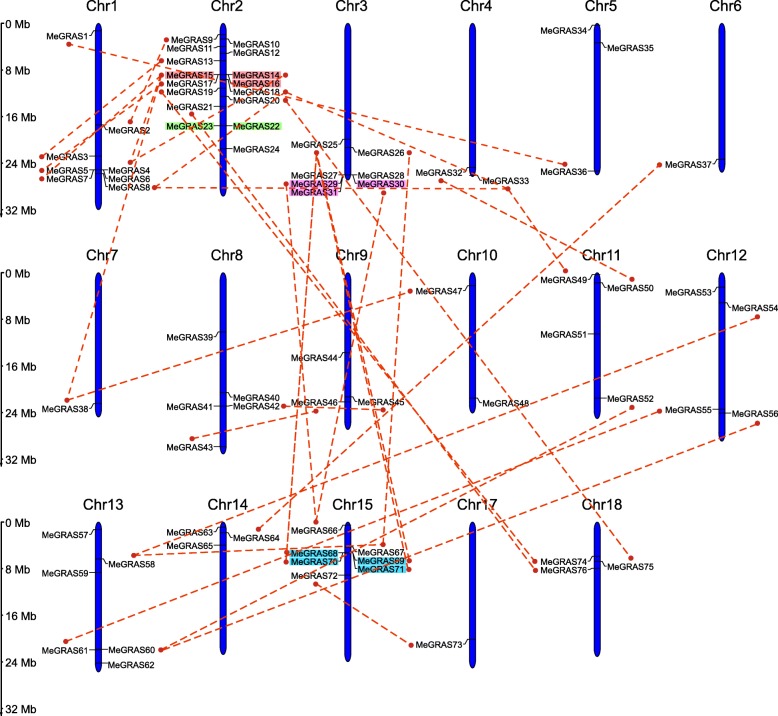
Positions of *GRAS* gene family members on cassava chromosomes. The duplicated *MeGRAS* genes are connected with the red lines. Tandem duplicated genes are indicated with a different-coloured background

All of the *MeGRAS* genes were unevenly distributed on the cassava chromosomes except *MeGRAS77* (Fig. 3). There were no *MeGRAS* members mapped onto Chr16. Chr2 contained the most *MeGRAS* genes (*n* = 16; 21.05%), followed by Chr1 (*n* = 8; 10.53%) and then both Chr3 and Chr15, each of which had seven members. In addition, six *MeGRAS* genes were distributed on Chr13, and five genes were distributed on Chr8. Four *MeGRAS* genes were found on both Chr11 and Chr12, three *MeGRAS* genes were distributed each on Chr5, Chr9, Chr14, and Chr18, and two *MeGRAS* genes were located on both Chr4 and Chr10. Only one *GRAS* gene was distributed on Chr6, Chr7, and Chr17. Notably, many *MeGRAS* genes were concentrated at both ends of the chromosomes.

**Fig. 4:**
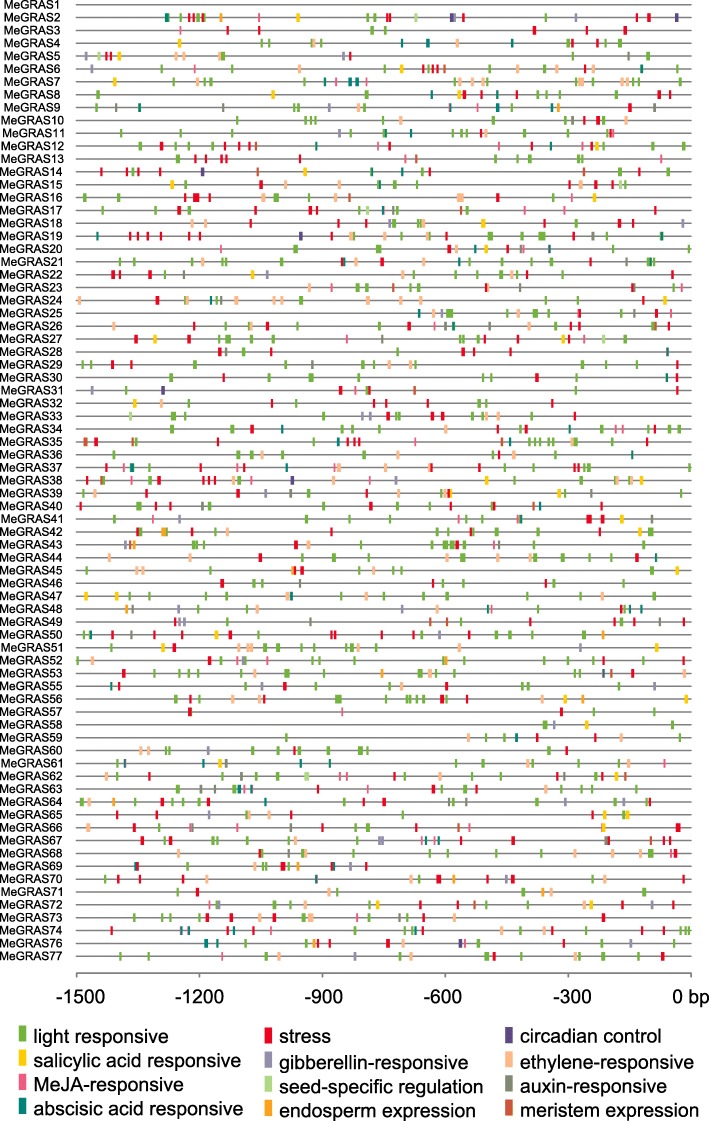
Predicted *cis*-elements in the promoter regions of *MeGRAS* genes. The number at the bottom indicates the nucleotides to the translation initiation codon, ATG

Gene duplication plays an important role in the occurrence of novel functions and gene family expansion; therefore, the duplication events of the *MeGRAS* gene within the cassava genome were analysed. As shown in Figs. 3 and 4, two groups of tandem duplicated genes (*MeGRAS14/15/16* and *MeGRAS22/23*) were located on Chr2, while two other groups of tandem duplicated genes (*MeGRAS29/30/31* and *MeGRAS68/69/70/71*) were located on Chr3 and Chr15, respectively. In addition, 34 pairs of *MeGRAS* genes were identified as being segmental duplications (Fig. 3).

### Analysis of *Cis*-elements in the *MeGRAS* promoters

The *cis*-elements were scanned in the promoter regions (1.5 kb upstream of the translation start site) of *MeGRAS* to better understand potential regulatory mechanisms of *MeGRAS* genes (Fig. 4). These *cis*-elements could be divided into four groups: 1) light responsive elements; 2) associated with defense and stress, such as drought, low-temperature, wounding and hypoxia; 3) related to plant hormone responses, such as ABA, MeJA, GA, auxin, salicylic acid and ethylene; and 4) involved in temporal and spatial gene expression, such as meristem, endosperm and seed. The identified motifs showed that *MeGRAS* may be regulated by various *cis*-elements within the promoter during growth.

### Interaction network of GRAS proteins in cassava

**Fig. 5:**
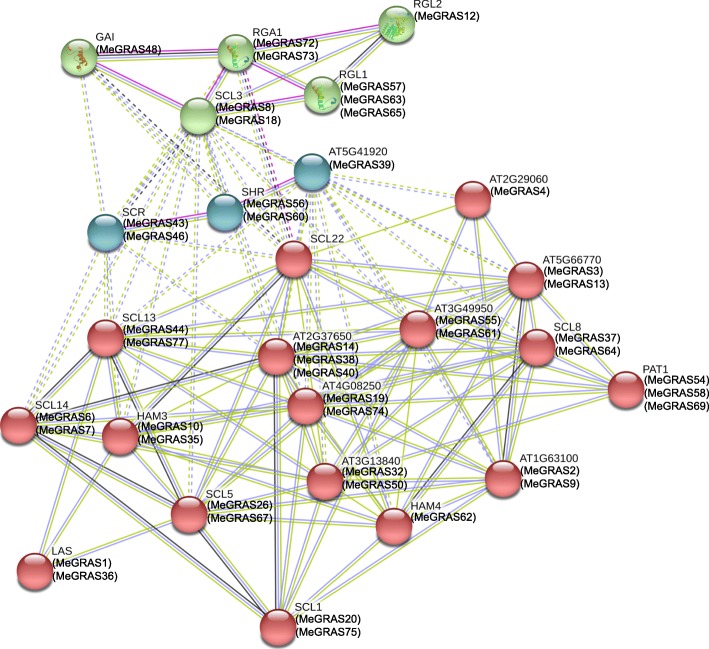
Functional interaction network of MeGRAS proteins in cassava according to orthologues in *Arabidopsis*

To understand the interactions of the MeGRAS proteins further, an interaction network was constructed via STRING software on the basis of the orthologues in *Arabidopsis*. The orthologous proteins with the highest bit score were considered STRING proteins, and only 46 MeGRAS proteins were selected because of the consideration of reliability (Fig. 5). In general, the MeGRAS proteins of the SCL3 subfamily (MeGRAS8 and 18) and LISCL subfamily (MeGRAS14, 38, and 40) had more interaction partners than did members of the other subfamilies. These findings were consistent with the working mechanisms in consideration of the regulation of GA homeostasis by AtSCL3 proteins, which integrate other signalling pathways; however, this relationship needs to be confirmed [47]. These interaction networks may provide significant clues to understanding the functions of unknown proteins.

### Expression analysis of *MeGRAS* genes in different tissues

**Fig. 6:**
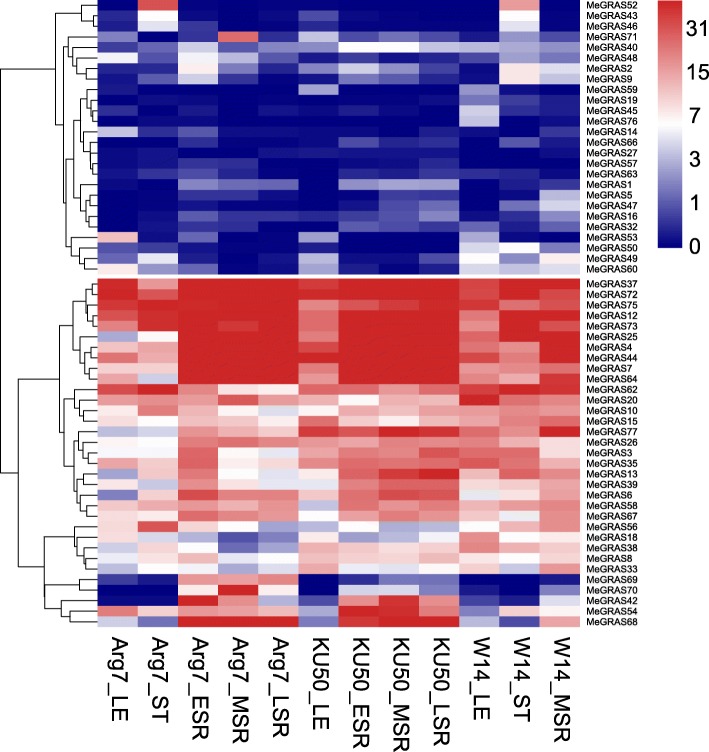
Expression profiles of *MeGRAS* genes in different tissues of three cassava varieties. LE, ST, ESR, MSR, and LSR represent leaves, stems, early-storage roots, middle-storage roots, and late-storage roots, respectively. FPKM values were used to generate the heat map with clustering. The scale represents the relative signal intensity of the FPKM values

Cumulative evidence has confirmed that GRASs play important roles in plant growth and development. To understand the function of *MeGRAS* genes in cassava better, the transcript levels of *MeGRAS* genes in different tissues, i.e., leaves, stems, early-storage roots, middle-storage roots, and late-storage roots, of the cultivated varieties Arg7 and KU50 and the wild subspecies W14 were examined via publicly available transcriptome data [2]. The fragments per kilobase of transcript per million mapped reads (FPKM) values of the *MeGRAS* genes are listed in Additional file 1: Table S7, and a heat map of the hierarchical clustering was generated to display the expression profiles of the *MeGRAS* genes (Fig. 6).

The expression of twelve *MeGRAS* genes (*MeGRAS21*, *22*, *23*, *24*, *28*, *29*, *30*, *41*, *51*, *55*, *61*, and *74*) was not detected in any of the analysed tissues, which is possibly due to differences in spatial and temporal expression patterns. With respect to the Arg7 variety, the expression of 63 out of 77 *MeGRAS* genes was detected in least one tissue, in which 26, 28, 34, 30, and 26 genes presented high transcript abundance (FPKM > 5) in the leaf, stem, early-storage root, middle-storage root and late-root storage tissues, respectively. With respect to the KU50 variety, the expression of 58 out of 77 *MeGRAS* genes was detected in at least one tissue, of which 26, 31, 29, and 30 genes presented high transcript abundance (FPKM > 5) in the leaf, early-storage root, middle-storage root and late-storage root tissues, respectively. With respect to the W14 variety, the expression of 61 out of 77 *MeGRAS* genes was detected in at least one tissue, of which 28, 33, and 31 genes presented high transcript abundance (FPKM > 5) in the leaf, stem, and middle-storage root tissues, respectively. Overall, with the exceptions of *MeGRAS25* and *MeGRAS64* in the leaf and stem tissues of Arg7, respectively, the expression levels of 15 *MeGRAS* genes (*MeGRAS37*, *72*, *75*, *12*, *73*, *25*, *4*, *44*, *7*, *64*, *62*, *20*, *15*, *26*, and *3*) from five subfamilies (PAT1, DELLA, LISCL, HAM, and SCL4/7) were high (FPKM > 5) in all of the tested tissues in the three varieties, suggesting key roles of these genes in tissue development.

Most *MeGRAS* genes exhibited similar expression profiles in the same tissues of Arg7, KU50, and W14, demonstrating that most *MeGRAS* genes play similar roles in tissue development in the three genotypes. However, a number of genes displayed different expression profiles. For example, the *MeGRAS33* transcript abundance was high (FPKM > 5) in the middle-storage roots of W14 but low in the middle-storage roots of Arg7 and KU50. In contrast, the *MeGRAS42* transcript abundance was high (FPKM > 5) in the middle-storage roots of Arg7 and KU50 but low in the middle-storage roots of W14. This phenomenon was also detected in other tissues. These findings indicate different roles of these genes in tissue development within different genotypes.

### Responses of *MeGRAS* genes to different abiotic treatments

To measure the transcript levels of *MeGRAS* genes under different abiotic stresses (drought, salt, cold, and H_2_O_2_) in different cassava varieties (D346, NZ199, and GR891), fifteen *MeGRAS* genes from different subfamilies were subjected to qRT-PCR.

**Fig. 7:**
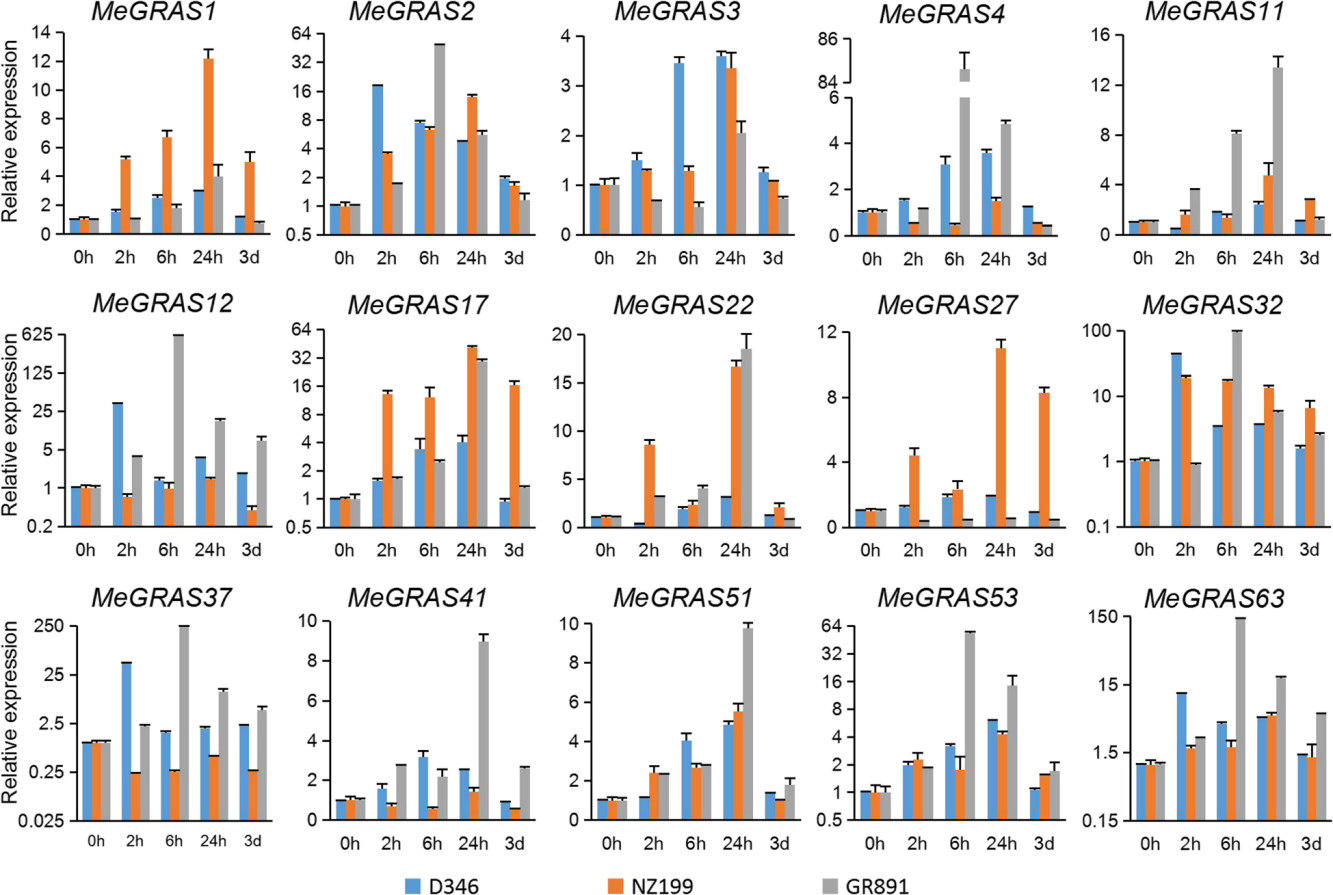
Expression of *MeGRAS* genes in response to drought treatment in the cassava varieties D346, NZ199, and GR891

Under drought treatment (Fig. 7), the expression of most *MeGRAS* genes was induced in the three cassava varieties. The expression of five *MeGRAS* genes (*MeGRAS1*, *3*, *11*, *17*, and *51*) peaked at 24 h and decreased after 3 d in the three cassava varieties. The expression of seven *MeGRAS* genes (*MeGRAS2*, *4*, *12*, *32*, *41*, *53*, and *63*) first increased but then decreased in D346 and tended to be relatively consistent in NZ199 and GR891, but the peaks differed. The expression of these seven *MeGRAS* genes was highly induced in GR891. The expression of *MeGRAS27* was clearly upregulated in both D346 and NZ199 but downregulated in GR891, whereas that of *MeGRAS37* clearly increased in both D346 and GR891 but decreased in NZ199. The expression of the *MeGRAS1* and *MeGRAS27* was higher in NZ199 than in D346 and GR891 under drought treatment.

**Fig. 8:**
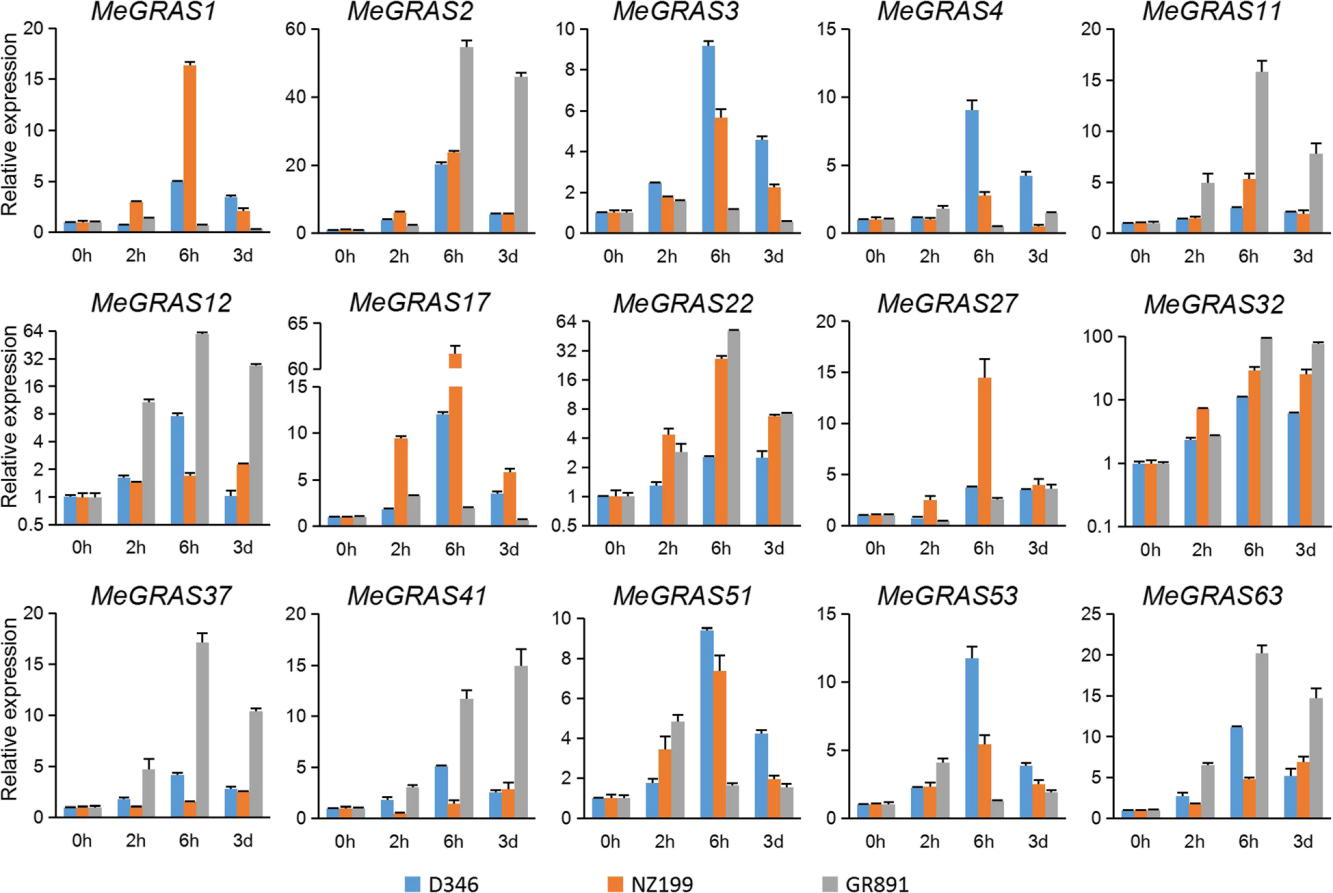
Expression of *MeGRAS* genes in response to salt treatment in the cassava varieties D346, NZ199, and GR891

Under salt treatment (Fig. 8), the expression of most *MeGRAS* genes first increased but then decreased in the three cassava varieties. The expression of four *MeGRAS* genes (*MeGRAS2*, *11*, *22*, and *32*) peaked at 6 h but decreased after 3 d in the three cassava varieties. The expression of four other *MeGRAS* genes (*MeGRAS1*, *3*, *17*, and *51*) peaked at 6 h in both D346 and NZ199, whereas it rapidly peaked at 2 h in GR891. The expression of three *MeGRAS* genes (*MeGRAS12*, *41*, and *63*) in NZ199 and three other *MeGRAS* genes (*MeGRAS27*, *37*, and *41*) in GR891 tended to increase. The expression of five *MeGRAS* genes (*MeGRAS32*, *12*, *2*, *22*, and *63*) was highly induced in GR891 under salt treatment, and the expression of two *MeGRAS* genes (*MeGRAS17* and *1*) was also highly induced in NZ199.

**Fig. 9:**
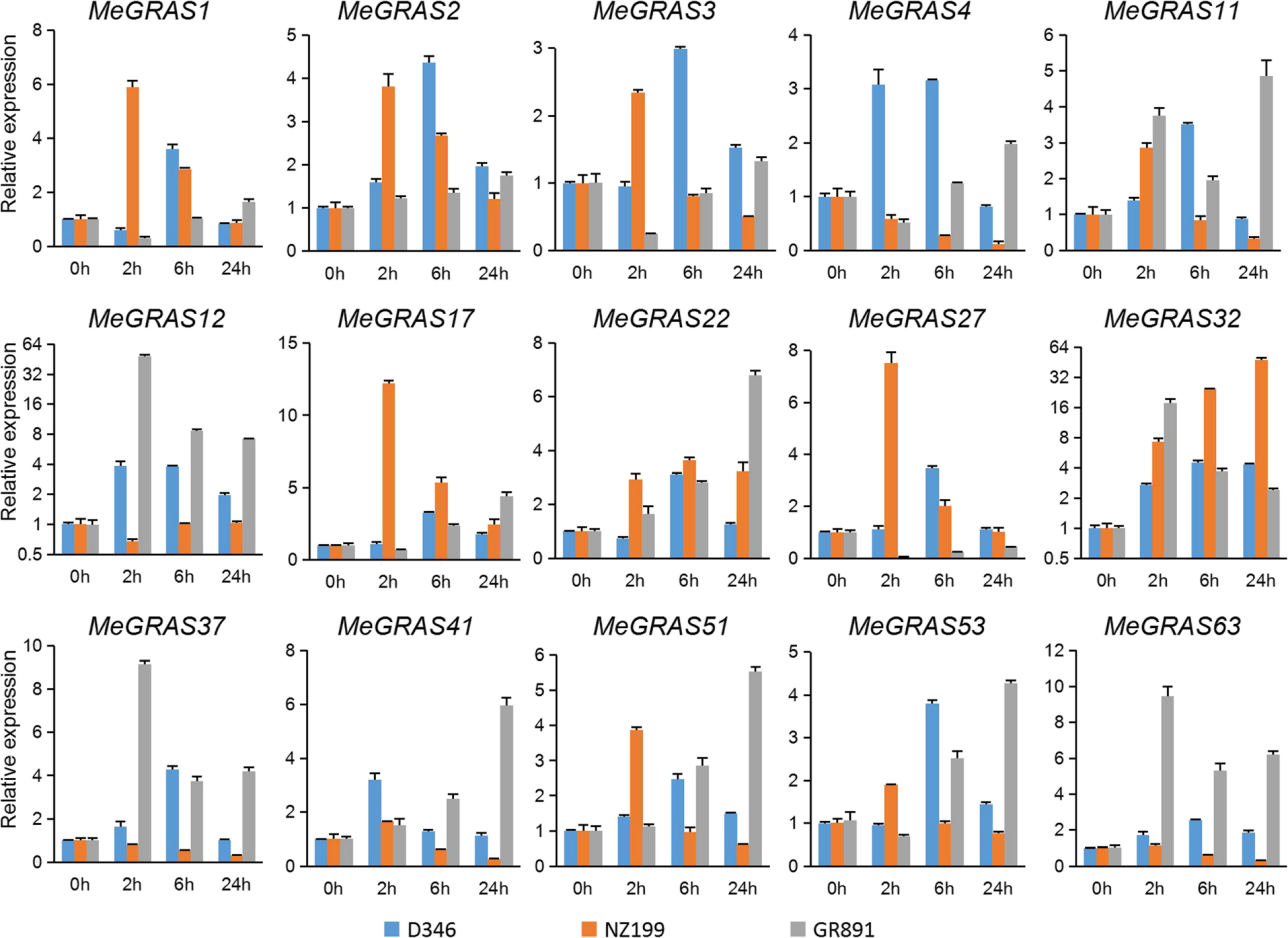
Expression of *MeGRAS* genes in response to cold treatment in the cassava varieties D346, NZ199, and GR891

Under cold treatment (Fig. 9), the expression of eight *MeGRAS* genes (*MeGRAS2*, *3*, *11*, *17*, *27*, *37*, *51*, and *53*) first increased but then decreased in both D346 and NZ199, peaking at 6 h and 2 h, respectively. The expression of five *MeGRAS* genes (*MeGRAS1*, *3*, *4*, *17*, and *53*) first decreased but then increased in GR891, reaching the lowest point at 2 h, and the expression of four *MeGRAS* genes (*MeGRAS2*, *22*, *41*, and *51*) was upregulated in GR891. *MeGRAS12* was the most highly induced gene in GR891, and *MeGRAS32* was highly induced in NZ199 under cold treatment.

**Fig. 10:**
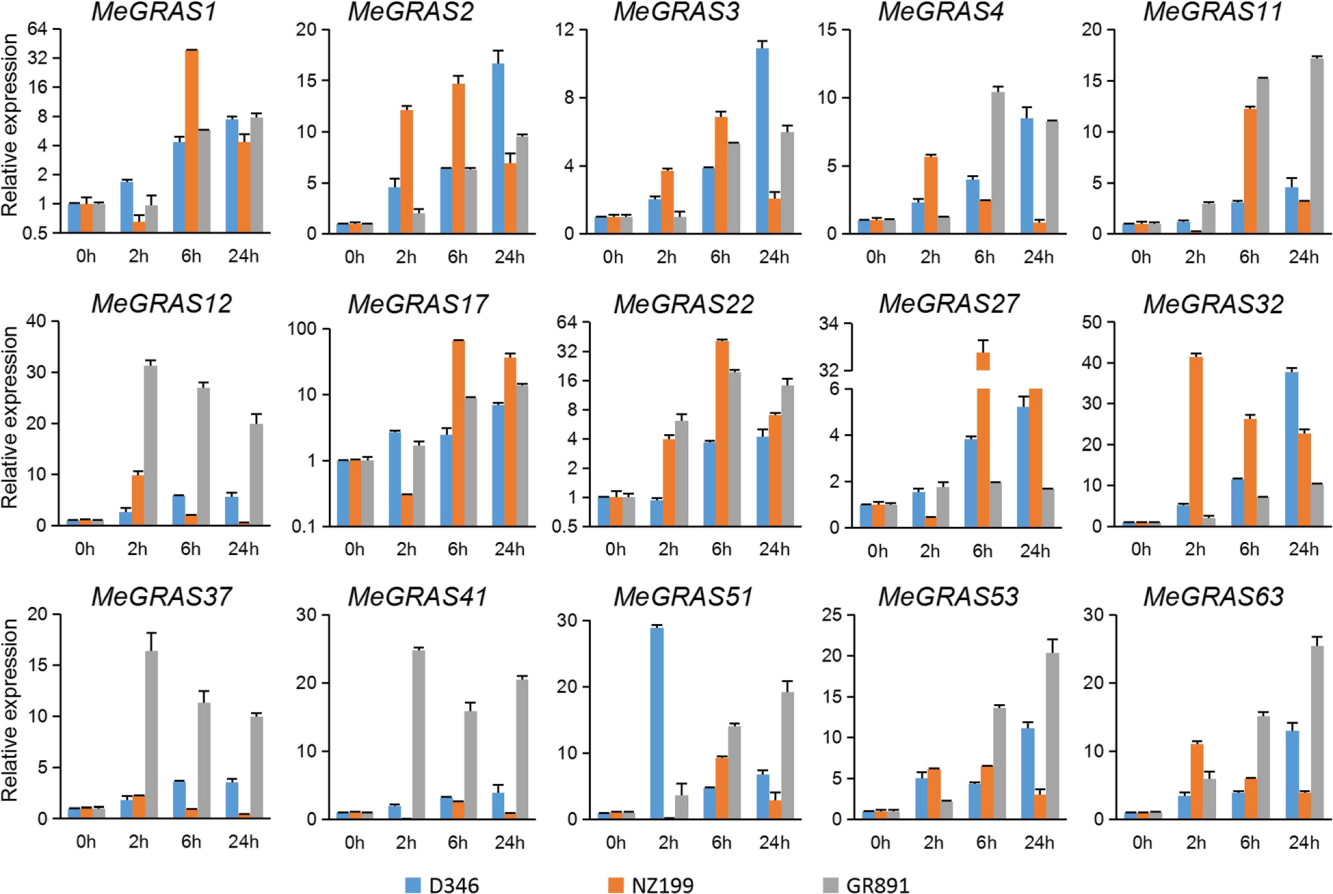
Expression of *MeGRAS* genes in response to H_2_O_2_ treatment in cassava varieties D346, NZ199, and GR891

Under H_2_O_2_ treatment (Fig. 10), the expression of nine *MeGRAS* genes (*MeGRAS1*, *2*, *3*, *4*, *11*, *27*, *32*, *41*, and *63*) was upregulated in D346, and that of nine *MeGRAS* genes (*MeGRAS1*, *2*, *3*, *11*, *17*, *32*, *51*, *53*, and *63*) was upregulated in GR891. The expression of nine *MeGRAS* genes (*MeGRAS2*, *3*, *4*, *12*, *22*, *32*, *37*, *53*, and *63*) first increased but then decreased in NZ199; the expression of five of those genes (*MeGRAS4*, *12*, *32*, *37*, and *63*) rapidly peaked at 2 h, and the expression of the other four *MeGRAS* genes (*MeGRAS2*, *3*, *22*, and *53*) peaked at 6 h. The expression of seven *MeGRAS* genes first increased but then decreased in GR891; the expression of two of them (*MeGRAS2* and *37*) rapidly peaked at 2 h, whereas the expression of three others (*MeGRAS4*, *22*, and *27*) peaked at 6 h. The expression of five *MeGRAS* genes (*MeGRAS17*, 3*2*, *22*, *1*, and *17*) was highly induced in NZ199, and that of four *MeGRAS* genes (*MeGRAS12*, *41*, *63*, and *53*) was highly induced in GR891. Last, the expression of the *MeGRAS32* and *51* genes was highly induced in D346 under H_2_O_2_ treatment.

## Discussion

GRAS transcription factors play vital roles in regulating the growth, development and stress responses of plants. However, the prevalence and functional diversity of the members of the GRAS family in cassava have not been comprehensively investigated. In the present study, the *GRAS* gene family in cassava was thoroughly analysed. We explored the features of *MeGRAS* genes, including their phylogenetic classification, gene structure, chromosomal distribution, *cis*-elements, expression profile, and responses to various stresses. The results enable us to research the evolution of the GRAS family and hypothesize about the potential functions of unknown genes.

In the present study, a total of 77 *GRAS* genes were identified in cassava. This number is lower than that in *Malus domestica* (127) [28], *Populus* (106) [20], and maize (86) [27] but higher than that in *Arabidopsis* (34) [20], rice (60) [20], Chinese cabbage (48) [19], *Prunus mume* (46) [21], tobacco (53) [22], castor (48) [23], grapevine (52) [24], *Medicago truncatula* (68) [25, 26], pepper (50) [29], and tea plant (52) [30]. This variation in *GRAS* gene numbers might be related to gene duplication events or genome size [24]. Four groups of tandem duplicated *MeGRAS* genes and 34 pairs of segmental duplicated *MeGRAS* genes were detected in this study. It appears that segmental duplication contributes more to cassava GRAS expansion than does tandem duplication. The *MeGRAS* genes were located on nearly all of the chromosomes except Chr16 and were unevenly distributed, with the “hot regions” on Chr2 (16 members). Consistent with that which occurs in other species such as *Medicago truncatula* [25], tomato [48], *Arabidopsis*, rice and *Populus* [20], most *GRAS* genes in cassava lack introns (53.2%) or have only a single intron (44.2%). The high proportion of intronless genes in the GRAS gene family suggests the close evolutionary relationship of GRAS members. Intronless genes have also been found in other large gene families, such as the DEAD-box RNA helicase [49] and F-box transcription factor families [50]. Although intronless genes are archetypical in prokaryotic genomes, one study [51] showed that plant *GRAS* genes originated from the prokaryotic genomes mainly by horizontal gene transfer as well as by duplication events throughout their evolution. This phenomenon may explain the formation of a substantial number of intronless *GRAS* genes.

The 77 MeGRAS proteins were divided into 14 subfamilies on the basis of their sequence homology and classification from *Arabidopsis* and rice [20]. It is noteworthy that some GRAS proteins considered to be species-specific in previous publications have homologs in cassava. For example, nine cassava *MeGRAS* genes (*MeGRAS22*, *23*, *27*, *28*, *29*, *30*, *31*, *34*, and *66*), together with *PtGRAS20* (belong to “Pt20” subfamilly) and *RcGRAS27* (29,889.m003282; belong to “Rc_GRAS” subfamily) which was previously regarded as species-specific subfamily [20, 23]. They were also clustered with tomato *SlGRAS22*, which was clustered with pepper-specific “Ca_GRAS” subfamily [29]. To summarize, all GRAS genes from “Pt20”, “Rc_GRAS” and “Ca_GRAS” subfamilies came from a same subfamily. Three (*MeGRAS57*, *63*, and *65*), two (*MeGRAS21* and *76*) and one (*MeGRAS51*) cassava *GRAS* genes, were clustered into “Os4”, “Os19” and “Os43” subfamily, respectively, which were previously reported as rice-specific protein subfamilies [20]. These four subfamilies did not include any *Arabidopsis* genes, implying lineage-specific gene loss in *Arabidopsis*. During evolution, other plant species may have lost these species-specific *GRAS* genes. Another possibility is that they became very specialized during the course of evolution. Analysis of the conserved motifs of the cassava proteins further corroborates the categorization of the MeGRAS family. Conserved motifs have been found within the GRAS domain regions and may have important functions. Although the conserved motifs were similar for all of these MeGRAS proteins, there were some differences in physicochemical features, which were also found among MeGRAS members. These differences may be due to the aa discrepancies within the regions of non-conserved MeGRAS members, suggesting that MeGRAS proteins function differently in their microenvironments [48].

Expression profiles of various tissues of different varieties with far affinity (wild subspecies and cultivated varieties) have been analysed extensively with respect to the functional characterization of *MeGRAS* genes. The RPKM data revealed no expression of 12 *MeGRAS* genes among five subfamilies (HAM, SHR, Os19, Os43, and Pt20) in any tissue but high expression of 15 *MeGRAS* genes (FPKM > 5) from 5 subfamilies (PAT1, DELLA, LISCL, HAM, and SCL4/7) in every tested tissue of these 3 varieties. There are differences in the expression patterns among these tissues, as demonstrated previously in Chinese cabbage [19], *Prunus mume* [21], and pepper [29]. Important roles have been inferred from high expression levels of *MeGRAS* genes. For example, *MeGRAS 73*, *72,* and *12* of the DELLA subfamily are highly important with respect to the control of various signalling hubs. Moreover, 7 *MeGRAS* genes (*MeGRAS 75*, *64*, *44*, *37*, *26*, *25* and *20*) from the PAT1 subfamily are expressed in a wide variety of tissues; these genes are probably involved in various developmental processes via phytochrome signalling regulatory systems, as is the case in *Arabidopsis* [13, 40]. Together, these results indicate that *GRAS* genes might have undergone sub-functionalization or neo-functionalization.

Harmful environmental conditions can cause substantial damage to the growth and development of cassava. *GRAS* genes may play key roles in plant responses against abiotic stresses [23, 25, 30, 48]. A *GRAS* gene in *poplar*, *PeSCL7*, is considered beneficial for engineering salt- and drought-tolerant trees [52], and overexpression of the *Brassica napus BnLAS* gene in *Arabidopsis* increases drought tolerance [53]. DELLA proteins are related to the response to many abiotic stresses, i.e., nitric oxide, cold, and phosphate starvation [54–56]. In the present study, expression analysis revealed that the expression of most GRAS genes in cassava is affected by various stress treatments (drought, salt, cold, and oxidative stress), suggesting that *MeGRAS* genes play crucial roles in the response to abiotic stress. Some expression trend differences occurred among the varieties D346, NZ199, and GR891. For example, under H_2_O_2_ treatment, the expression of *MeGRAS4* first increased but then decreased in NZ199 and GR891, but it peaked at 2 h in NZ199 and at 6 h in GR891; on the other hand, the expression of *MeGRAS4* increased in D346 but did not peak within 24 h. These different trends of *MeGRAS* gene expression may be related to different responses to abiotic stress in the three varieties.

This comprehensive study provides a basis for further investigation of *GRAS* genes in cassava and could also have potential value for the genetic improvement of cassava as well as other related species.

## Methods

### Identification of *GRAS* genes in cassava

The latest versions of the genome annotations of cassava (*Manihot esculenta* v6.1) were downloaded from the Phytozome v12 database (https://phytozome.jgi.doe.gov/). Annotated protein databases were scanned using HMMER 3.0 (http://hmmer.org/) with the Hidden Markov model (HMM) of the GRAS domain (PFD03514), which was downloaded from Pfam (http://pfam.xfam.org/). On the basis of the proteins acquired through the GRAS HMM, a high-quality protein set was aligned (E-value <1e^− 20^) and used to construct a cassava-specific GRAS HMM using hmmbuild in HMMER 3.0. This new cassava-specific HMM was used to select all of the proteins with an E-value lower than 1e^− 5^. In addition, all of the OsGRAS and AtGRAS proteins were used as queries to explore the cassava database via the default parameters. With the application of Pfam database and the Conserved Domain Database (CDD, https://www.ncbi.nlm.nih.gov/Structure/cdd/wrpsb.cgi), only those sequences having a full-length GRAS domain were selected as MeGRAS proteins and used for the subsequent analyses.

ProtParam (http://web.expasy.org/protparam/) was used for the prediction of the physical and chemical features of MeGRAS proteins. To verify the subcellular localization of the identified MeGRAS proteins, WoLF PSORT was used to predict the protein sequences (https://wolfpsort.hgc.jp/). TMHMM Server v2.0 (http://www.cbs.dtu.dk/services/TMHMM/) was used for predicting the transmembrane helices in the proteins.

### Phylogenetic analysis of *GRAS* genes

This research investigated the GRAS proteins of cassava, *Arabidopsis*, rice, castor bean, *Populus*, tomato, and tea plant. *Arabidopsis* and rice are the most commonly used model plant species for researching genetic correlations. One *GRAS* gene was selected from each subfamily of castor bean, *Populus*, tomato, and tea plant for a better classification of *MeGRAS* genes. Additional file 1: Table S5 lists the gene IDs of the GRAS members. An unrooted phylogenetic tree was constructed via the Maximum Likelihood method with 10,000 bootstrap replicates using MEGA-X (https://www.megasoftware.net/). The cassava GRAS members were further classified into various subcategories on the basis of the well-established division in other species [18, 20, 23, 29, 48].

### Protein conserved motifs and gene structure analysis

The MEME program was used to identify the conserved motifs. The search also involved the default parameters, except for the maximum number of motifs, which was set to 20. The gene structure of the cassava GRASs was determined via the Gene Structure Display Server (GSDS) 2.0 (http://gsds.cbi.pku.edu.cn/) program.

### Chromosomal mapping and gene duplication analysis

Every *GRAS* gene was matched with the chromosomes of cassava on the basis of the genome annotations of cassava. MapGene2Chromosome (http://mg2c.iask.in/mg2c_v2.0/) was used to draft the map. Gene duplication was explored for *MeGRAS* genes according to the method described in maize [27]; this method involved 1) the alignment of the entire covered protein length, which is > 80% of the longest gene, 2) > 80% identity of the aligned region, and 3) the counting of only one duplication event for tightly linked genes. If there were 5 or fewer than 5 genes separated by two homologous genes, they were labelled as tandem duplications. However, when there were more than 5 genes separating these two genes or there were distributions across various chromosomes, they were referred to as segmental duplications.

### Promoter *Cis*-elements analysis

The upstream of 1.5 Kb were used for *cis*-elements in the promoters of the candidate *MeGRAS* genes. PlantCARE software (http://bioinformatics.psb.ugent.be/webtools/plantcare/html/) was used for searching regulatory elements.

### Prediction of the MeGRAS protein-protein interaction network

To illustrate the correlations between MeGRASs further, interologues of *Arabidopsis* were used to predict the protein-protein interaction network. STRING software was used to construct the functional interaction networks of proteins, with the confidence parameter set at 0.15 [57].

### Expression analysis of *MeGRAS* genes in different tissues

Transcriptome data available online were used to explore the expression profiles of *MeGRAS* genes in various tissues in various cassava varieties [2] (Additional file 1: Table S8 lists the accession numbers). Tissues from the leaves, stems, late-storage roots, middle-storage roots, and early-storage roots of a wild subspecies (W14) and two cultivated varieties (KU50 and Arg7) were sampled to investigate the expression profiles of *MeGRAS* genes. RPKM values were subsequently calculated to evaluate the gene expression.

### Cassava plant preparation and stress treatments

All of the studied plants were obtained from a glasshouse at Guangxi University (Nanning, China) between April and July 2018. Cassava stem segments were transplanted into individual pots. The plants were then watered regularly. Three-month-old plants of three cassava varieties (D346, NZ199, and GR891) whose resistance to stress was different were subjected to different abiotic stress treatments, which included 20% polyethylene glycol (PEG) 6000 for 2, 6, and 24 h and 3 d; 300 mM NaCl for 2, 6, and 24 h; cold (4 °C) for 2, 6, and 24 h; and 10% H_2_O_2_ for 2, 6, and 24 h. Every sample comprised three independent biological replications.

### RNA isolation and qRT-PCR expression analysis

An RNA extraction kit (Huayueyang, China) was used to extract the mRNA from the leaves after each treatment. cDNA was used for the reverse transcription of 1 μg of total mRNA via a cDNA synthesis kit (Takara, Japan). Primer 5.0 was used to design the primers used (Additional file 1: Table S9). For normalization, the *MeActin* gene was used, serving as an endogenous control. The reaction mechanism of PCR contained 0.5 μL of primers, 1 μL of template cDNA and 5 μL of 2X ChamQ SYBR qPCR Master Mix (Vazyme, China). Afterwards, ddH_2_O was added to reach a final volume of 10 μL. The protocol was as follows: 95 °C for 30 s followed by 40 cycles of 95 °C for 10 s, 55 °C for 10 s, and 72 °C for 20 s. Each reaction was performed three times, and the 2^-ΔΔCT^ method [58] was used to calculate the relative gene expression levels.

## Conclusions

In conclusion, 77 *GRAS* gene family members from the cassava genome were characterized and classified into 14 subfamilies on the basis of phylogenetic relationships. The gene structure and motif compositions of the proteins were considerably conserved within the same subgroup. Duplication events, particularly segmental duplication, were identified as the main driving force for *GRAS* gene expansion in cassava. Global expression analysis revealed that the expression profiles of the *MeGRAS* genes were similar or distinct within different tissues among different varieties. The expression patterns of *MeGRAS* genes in response to abiotic stress suggested that these genes possibly have multiple functions. Overall, our study is the first comprehensive characterization of *GRAS* genes in cassava. These data provide a foundation for elucidating the GRAS-mediated molecular mechanism underlying plant growth and development as well as stress biology. This study could serve as a reference for future functional investigations and molecular breeding of cassava.

## Supplementary information

**Additional file 1: Table S1.** List of the GRAS protein sequences in cassava. **Table S2.** A catalog of MeGRAS proteins with their HMM profiles. **Table S3.** Protein property and subcellular localization of MeGRAS proteins. **Table S4.** Secondary structure of amino acid sequences in cassava. **Table S5.** *GRAS* genes used in *Arabidopsis*, rice, castor bean, *Populus*, tomato, and tea plant. **Table S6.** The structural features of motif 1–20. **Table S7.** The expression data of the cassava *GRAS* genes in different tissues. **Table S8.** The accession number of transcriptomic data in NCBI. **Table S9.** Sequences of primers used in qRT-PCR.

## Data Availability

The RNA sequencing (RNA-seq) data of each *MeGRAS* gene were obtained from previous studies in different tissues of different cassava varieties [2].

## Abbreviations

Aa: Amino acid
Chr: Chromosome
FPKM: Fragments per kilobase of transcript per million mapped reads
